# Application-oriented fundamental research on concrete and reinforced concrete structures: selected findings from an Austro-Chinese research project

**DOI:** 10.1007/s00707-020-02639-1

**Published:** 2020-03-12

**Authors:** Eva Binder, Hui Wang, Jiao-Long Zhang, Thomas Schlappal, Yong Yuan, Herbert A. Mang, Bernhard L. A. Pichler

**Affiliations:** 1 College of Civil Engineering, Tongji University, 1239 Siping Road, Shanghai, China; 2 Institute for Mechanics of Materials and Structures, TU Wien - Vienna University of Technology, Karlsplatz 13/202, 1040 Vienna, Austria; 3 Department of Transportation Engineering, School of Naval Architecture, Ocean and Civil Engineering, Shanghai Jiao Tong University, 800 Dongchuan Road, Shanghai, China

## Abstract

In this paper, the significance of application-oriented fundamental research on concrete and reinforced concrete structures for progress regarding practical applications to structural design is addressed based on four examples. They were treated in a joint research project of Vienna University of Technology and Tongji University. The first topic refers to sudden heating or cooling of concrete structures, the second one to high-dynamic strength of specimens made of cementitious materials, the third one to structural analysis of segmental tunnel rings used in mechanized tunneling, and the fourth one to serviceability and ultimate limit states of concrete hinges used in integral bridge construction. The first two topics deal with exceptional load cases. Results from the fundamental research call for improvements of state-of-the-art simulation approaches used in civil engineering design. The last two topics refer to reinforced concrete hinges used in mechanized tunneling and integral bridge construction, respectively. Integrative research has led to progress regarding the verification of serviceability and ultimate limit states. In all four examples, results from fundamental research are used to scrutinize state-of-the-art approaches used in practical structural design of civil engineering structures. This allows for identifying interesting directions for the future development of design guidelines and standards.

## Introduction

This paper is focused on four examples of fundamental research carried out in the field of engineering mechanics of concrete and reinforced concrete structures. The problems concerned were treated in a recently completed research project, called “Bridging the Gap by Means of Multiscale Structural Analyses” [1]. This project was a cooperative effort of Vienna University of Technology, in Austria, Europe, and Tongji University, in Shanghai, China. It focused on the added value resulting from the use of modern multiscale material models for concrete in the framework of structural analysis of reinforced concrete structures. The first two examples refer to exceptional load cases, and the remaining two to the regular service of civil engineering infrastructure.

Sudden heating or cooling is the topic of the first example. A multiscale poromechanics model was used to explain why the macroscopic thermal expansion coefficient of mature cement paste is a nonlinear function of the internal relative humidity [2]. At the macrostructural scale, thermal eigenstrains, resulting from transient heat conduction, were analyzed [3, 4]. Herein, the question is discussed whether or not it is possible to define equivalent temperature gradients, such that a structural analysis can be carried out with commercial simulation software, such as recommended by the state-of-the-art guideline [5]. In addition, durability issues, resulting from recurrent cycles of temperature and relative humidity, are discussed.

The high-dynamic strength of cementitious materials is the topic of the second example. Fundamental research in this area is based on the theory of propagation of stress waves and cracks through linear-elastic, isotropic media. An engineering-mechanics approach was used to develop a model for the high-dynamic compressive and tensile strength of specimens made of cement pastes, mortars, and concretes [3, 6–8]. Herein, the question is discussed whether or not it is possible to analyze concrete structures subjected to dynamic loading by means of commercial simulation software for quasi-static analysis, simply by introducing higher values of the strength.

Structural analysis of real-scale tests of segmental tunnel rings is the topic of the third example. Bearing capacity tests were analyzed with the help of transfer relations in the form of analytical solutions of the first-order theory of thin circular arches. In the developed hybrid analysis method, transfer relations and measured relative rotations of neighboring segments were combined [9, 10]. Herein, the discussion focuses on the convergences, in the vertical and the horizontal direction, governing the serviceability limits, and on the development of plastic hinges at the interfaces, defining the bearing capacity of segmental tunnel rings.

Recommendations for verification of serviceability and ultimate limit states of reinforced concrete hinges are the topic of the fourth example. An engineering-mechanics model was established to determine acceptable relative rotations as a function of the normal force transmitted across the neck [11, 12]. The usefulness of corresponding dimensionless diagrams was checked by means of experimental data. Herein, the discussion focuses on similarities of serviceability limit states of concrete hinges in integral bridge construction and mechanized tunneling and on differences between the two.

The main aim of the present paper is to use results from fundamental research in the civil engineering science as the basis for scrutinizing state-of-the-art approaches used in practical structural design of civil engineering structures. This allows for identifying interesting directions for the future development of design guidelines and standards.

The paper is structured as follows: one section each is dedicated to the aforementioned four examples, see Sects. 2–5. Overall conclusions are drawn in Sect. 6.

## Multiscale thermoelastic analysis of concrete and concrete structures

Concrete structures, such as pavement plates made of plain concrete and tunnel segments made of reinforced concrete, are generally subjected to recurrent cycles of temperature and relative humidity. They may have to sustain exceptional loadings, such as sudden heating in case of fire disasters, or sudden cooling in case of hail showers. The resulting thermomechanical loading may lead to damage of these structures. Thus, it is a threat to their long-term durability and safety.

**Fig. 1:**
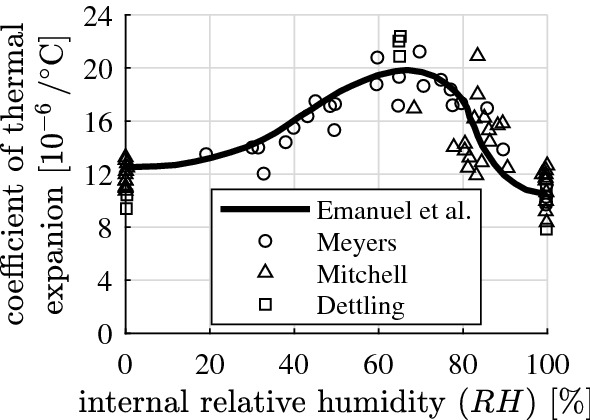
Dependence of the thermal expansion coefficient of cement paste on the internal relative humidity: experimental results of Meyers [13], Mitchell [14], and Dettling [15]; curve obtained by Emanuel and Hulsey [16]

The thermal expansion coefficient of concrete governs the thermal deformations and stresses of concrete structures subjected to thermal loading. This coefficient is a nonlinear function of the internal relative humidity *RH* of the material, because the thermal expansion coefficient of the cement paste is an asymmetrical bell-shaped function of *RH*, see Fig. 1. Its maximum value occurs at $RH \approx 65\%$, which is virtually twice as large as its minimum value at $RH = 100\%$. This provided the motivation for fundamental scientific research regarding the thermal expansion coefficient of cement paste and concrete.

At the structural scale of pavement plates, heat conduction mainly concerns the thickness direction. When it comes to transient heat conduction, the temperature and the associated thermal eigenstrains are time dependent. Their distributions over the thickness of a plate are nonlinear. However, there still exist state-of-the-art guidelines for quantification of thermal stresses of pavement plates recommending the assumption of a linear distribution of the temperature along the thickness of the plate, see, e.g., [5]. This provided the motivation for research regarding the contribution of the nonlinear part of the temperature profile to the thermal stresses.

### Results from recent fundamental research

This Subsection is organized in three parts. At first, a thermoporoelastic multiscale material model is used to provide insight into the question why thermal expansion coefficients of cementitious materials depend on the internal relative humidity. Subsequently, a structural analysis of a pavement plate subjected to solar heating followed by a suddenly starting hail shower is discussed. Finally, methods available for top-down quantification of stresses experienced by the concrete constituents (the cement paste, the aggregates, and the interfacial transition zones) are briefly summarized.

**Fig. 2:**
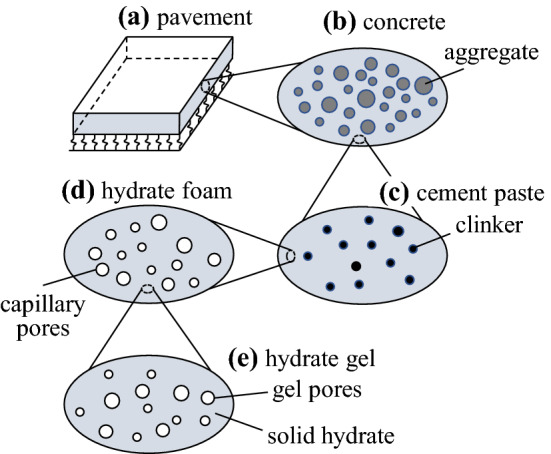
Multiscale representation of a concrete pavement plate, following [17]

Concrete is a hierarchically organized microheterogeneous material, see Fig. 2. The microstructure of concrete consists of aggregate inclusions embedded in a matrix of cement paste. The microstructure of cement paste consists of unhydrated cement clinker inclusions embedded in a matrix of hydrate foam. The microstructure of the hydrate foam consists of capillary pores embedded in a matrix of hydrate gel. Finally, the microstructure of the hydrate gel consists of nanoscopic gel pores embedded in a matrix of solid hydrates.

Subsequent homogenization of the four matrix-inclusion composites in Fig. 2 is used for bridging the scales from the nanoscopic level of the solid hydrates and the gel pores to the macroscopic scale of the concrete. Each representative volume element (RVE), occupying a volume of $V_{\text {RVE}}$, consists of a matrix phase with a volume of $V_m$ and an inclusion phase with a volume of $V_i$. Both material phases $k \in [m, \,i\,]$ exhibit a specific elastic stiffness, $\mathbb {C}_k$, a specific eigenstress, $\varvec{\sigma }_k^e$, and a specific volume fraction, $f_k$,

1 $$\begin{aligned} \forall \underline{x} \in V_{k} \,: \, \left\{ \begin{array}{l} \mathbb {C} (\underline{x}) = \mathbb {C}_k\\ \varvec{\sigma }^e (\underline{x}) = \varvec{\sigma }_k^e \end{array} \right. , \quad f_k = \frac{V_k}{V_{\text {RVE}}} \,, \quad k \in [\,m\,, \,i\,] \,. \end{aligned}$$

These three properties are discussed in the following paragraph.

The solid constituents, i.e., the solid hydrates (shyd), the unhydrated clinker grains (clin), and the aggregates (agg), are considered to be isotropic. Thus, their elastic stiffness can be described based on their bulk and shear moduli [2]. The gel and capillary pores, in turn, are filled by water or air. Thus, they are characterized by vanishing solid stiffnesses. The eigenstresses of the solid constituents, $\varvec{\sigma }_{k}^{e}$, are proportional to the thermal eigenstrains $\varvec{\varepsilon }_k^e$, i.e.,

2 $$\begin{aligned} \varvec{\sigma }_{k}^{e} = - \mathbb {C}_k:\varvec{\varepsilon }_k^e, \quad k \in [{\hbox {shyd}}; {\hbox {clin}}; {\hbox {agg}}]. \end{aligned}$$

The latter are proportional to the thermal expansion coefficients $\alpha _{k}$ and the temperature change $\varDelta T$,

3 $$\begin{aligned} \varvec{\varepsilon }_k^e = \alpha _{k} \, \varDelta T \, \varvec{1}, \quad k \in [{\hbox {shyd}}; {\hbox {clin}}; {\hbox {agg}}]. \end{aligned}$$

The pores are idealized as being connected and spherical, with radii *r* following exponential distributions:

4 $$\begin{aligned} \phi _{k}(r) = \frac{1}{R_{k}} \exp \left( - \frac{r}{R_{k}} \right) , \quad k \in [{\hbox {gpor}}; {\hbox {cpor}}] \end{aligned}$$

where *gpor* stands for gel pores and *cpor* for capillary pores. The characteristic radius $R_\mathrm{gpor}$ amounts to $\approx$2 nm, $R_\mathrm{cpor}$ to $\approx$12 nm, see [2]. As for homogenization, the eigenstresses of gel and capillary pores are proportional to the average effective pore pressures [18],

5 $$\begin{aligned} \varvec{\sigma }_k^e = -p_k \, \varvec{1}, \quad k \in [{\hbox {gpor}}; {\hbox {cpor}}]. \end{aligned}$$

$p_\mathrm{gpor}$ and $p_\mathrm{cpor}$ are obtained from averaging the effective pressures *p*(*r*), referring to individual pores with radius *r*, over pores of all sizes [2]:

6 $$\begin{aligned} p_k = \int _{0}^{\infty } p(r) \, \phi _{k}(r) \, \mathrm {d}r , \quad k \in [{\hbox {gpor}}; {\hbox {cpor}}]. \end{aligned}$$

The effective pressures of individual pores result from the pressure of the fluids filling the pores, either the liquid pressure $p_\ell$ or the gas pressure $p_g$, and from the surface tension prevailing at the interface between the pore and the surrounding solid, either that at solid-liquid interfaces, $\gamma ^{s \ell }$, or that at solid-gas interfaces, $\gamma ^{sg}$. Because of the latter contributions, the effective pressures are a function of the radius *r* of the pores,

7 $$\begin{aligned} p(r) = \left\{ \begin{array}{l} p_{g} - \frac{2 \, \gamma ^{s g}}{r - t} \quad \cdots \quad r > r_\mathrm{Kelvin}, \\ p_{\ell } - \frac{2 \, \gamma ^{s \ell }}{r - t} \quad \, \cdots \quad r \le r_\mathrm{Kelvin}, \end{array} \right. \end{aligned}$$

where *t* denotes the thickness of the layer of water adsorbed to the surface of the pores. The Kelvin radius $r_\mathrm{Kelvin}$ discriminates between water-filled and air-filled pores. It is a function of the temperature and the relative humidity [2]. Setting $p_g$ equal to zero and considering the Kelvin–Laplace equation, Young’s equation, and Berthelot’s state equation, Eq. (7) can be reformulated in terms of measurable quantities as well as the universal gas constant *R*, the absolute temperature *T*, and the molar volume of water $\nu _m$ as [2]

8 $$\begin{aligned} p(r) = \left\{ \begin{array}{l} -\frac{2 \, \gamma ^{\ell g}}{r - t} \quad \quad \quad \cdots \quad r > r_\mathrm{Kelvin}, \\ \ln (RH) \frac{R \, T}{\nu _m} \quad \cdots \quad r \le r_\mathrm{Kelvin}. \end{array} \right. \end{aligned}$$

The volume fractions of the solid constituents and the pores are quantified based on Powers’ hydration model [2]. They are functions of the initial composition of concrete, quantified in terms of the initial aggregate-to-cement mass ratio and the initial water-to-cement mass ratio, and of the maturity of concrete, quantified in terms of the hydration degree.

Bottom-up homogenization of the RVEs in Fig. 2 is carried out in a step-by-step fashion, using methods of continuum micromechanics [19]. The thermoelastic behavior of homogenized composites follows the generalized Hooke’s law as

9 $$\begin{aligned} {\varvec{\Sigma }}_{\hom } = \mathbb {C}_{\hom }:\mathbf {E}_{\hom } + {\varvec{\Sigma }}_{\hom }^e \,, \end{aligned}$$

where ${\varvec{\Sigma }}_{\hom }$ and $\mathbf {E}_{\hom }$ denote the macroscopic stress and strain, respectively. The homogenized stiffness, $\mathbb {C}_{\hom }$, and eigenstress, ${\varvec{\Sigma }}_{\hom }^e$, follow as

10 $$\begin{aligned} \mathbb {C}_{\hom }&= f_m \, \mathbb {C}_m : \mathbb {A}_m + f_i \, \mathbb {C}_i : \mathbb {A}_i \,, \end{aligned}$$

11 $$\begin{aligned} {\varvec{\Sigma }}_{\hom }^e&= f_m \, \varvec{\sigma }_m^e : \mathbb {A}_m + f_i \, \varvec{\sigma }_i^e : \mathbb {A}_i \,, \end{aligned}$$

with $\mathbb {A}_k$ standing for the strain concentration tensor of phase *k*. $\mathbb {A}_k$ are estimated by means of the Mori–Tanaka–Benveniste scheme [20]. Under consideration of macroscopic stress-free expansion, ${\varvec{\Sigma }}_{\hom }=0$, the macroscopic strain is, on the one hand, proportional to the homogenized eigenstress, $\mathbf {E}_{\hom }=-(\mathbb {C}_{\hom })^{-1}:{\varvec{\Sigma }}_{\hom }^e$. On the other hand, it is proportional to the homogenized thermal expansion coefficient, $\mathbf {E}_{\hom } = \alpha _{\hom } \, \varDelta T \, \varvec{1}$. Thus, $\alpha _{\hom }$ can be calculated.

**Fig. 3:**
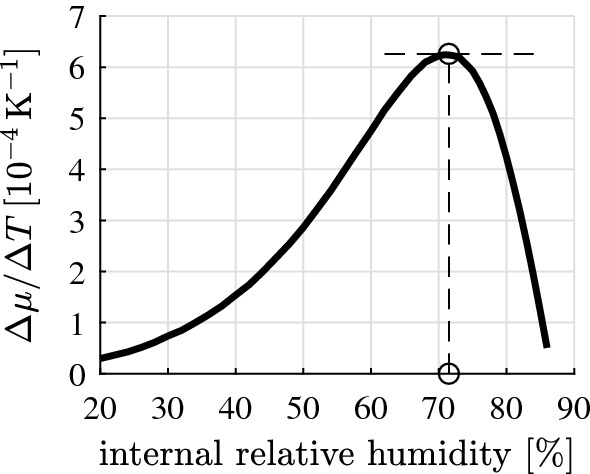
Specific water uptake/release of hydration products per temperature increase, $\varDelta \mu / \varDelta T$, identified in [2]

The described multiscale model allows for explaining the thermal expansion behavior of cement paste (Fig. 1), based on the water uptake/release characteristics of the nanoscopic solid hydrates, a formerly unknown material property, see Fig. 3. The water uptake/release coefficient $\varDelta \mu / \varDelta T$ is a bell-shaped function of the relative humidity prevailing in the air-filled pores before the temperature change $\varDelta T$. Thereby, $\varDelta \mu$ denotes the change of the mass of the hydrates resulting from the uptake/release of water, divided by the initial mass of the hydrates: $\varDelta \mu = \varDelta m_\mathrm{hyd} / m_\mathrm{hyd}$. The uptake/release of water is a rather subtle effect, but still large enough to explain the thermal expansion behavior of cement paste. Provided that the temperature of the material is increased, the solid constituents expand according to their thermal expansion coefficients *and* the solid hydrates release water. This results in a redistribution of water within the partially saturated pore network and in an increase of the Kelvin radius. Consequently, the internal relative humidity increases and the average effective pore underpressures decrease [2]. This manifests itself macroscopically as an additional poromechanical contribution to the macroscopic swelling of the cement paste. The opposite effects occur when cement paste is cooled down. The solid constituents shrink according to their coefficients of thermal expansion *and* the solid hydrates take up water, the Kelvin radius decreases, the internal relative humidity decreases, the average effective pore underpressures increase, and this manifests itself macroscopically as an additional poromechanical contribution to the macroscopic shrinkage of the cement paste. As regards the experimental validation of the described phenomena, the water uptake/release characteristics of the hydrates were observed recently based on proton nuclear magnetic resonance relaxometry tests of cement paste subjected to temperature changes [21].

**Fig. 4:**
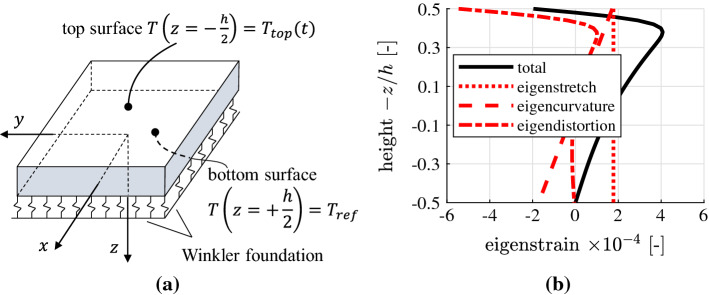
**a** Schematic illustration of a pavement subjected to solar heating followed by a hail shower, and **b** thermal eigenstrains and their decomposition into the eigenstretch, the eigencurvature, and the eigendistortion, 3 min after the start of the hail shower [4]

The homogenized thermomechanical properties of concrete [22] are used as input for the structural analysis of a pavement plate subjected to solar heating, followed by a suddenly starting hail shower [4]. The thickness of the plate amounts to 25 cm, the homogenized Young’s modulus of concrete to 32 GPa, and the homogenized coefficient of thermal expansion to $11.5\times 10^{-6}/^\circ \mathrm {C}$. At first, heat conduction in the thickness direction is analyzed. In the initial configuration, the plate is in an isothermal state at $17\,^\circ$C. As regards the boundary conditions, the temperature is set constant at the bottom of the plate, and the top surface is subjected to a prescribed temperature evolution. During the first 12 hours of the analysis, solar heating results in an increase of the surface temperature from 17 to $62\,^\circ$C. After that, a suddenly starting hail shower reduces the surface temperature instantaneously to $0\,^\circ$C. This surface temperature remains constant for several minutes. The described thermal loading results in transient heat conduction. This problem can be solved based on existing series solutions [4], providing quantitative access to the time-dependent and spatially nonlinear distributions of the temperature. The resulting thermal eigenstrains of concrete are equal to the product of the temperature changes and the homogenized thermal expansion coefficient. Thus, the spatial distribution of the thermal eigenstrains is also nonlinear, see Fig. 4 for the eigenstrains obtained 3 min after the start of the hail shower.

**Fig. 5:**
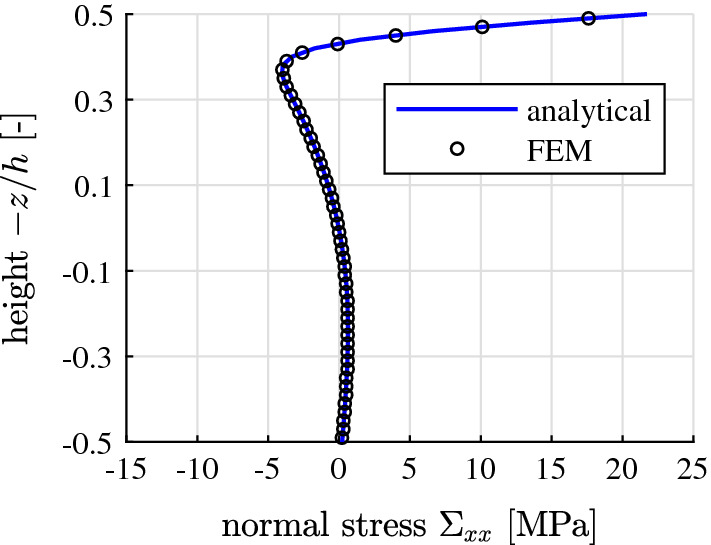
Normal stresses induced by the eigendistortions at the time instant 3 min after the start of the hail shower, comparison between the results of the analytical solution presented in [4] and a Finite Element simulation

The thermal stresses of the pavement plate are quantified using the theory of thin plates [4]. It is based on Kirchhoff’s normal hypothesis. It implies that the generators of thin plates remain straight. Thus, the total strains (i.e., the sum of the thermal eigenstrains and the stress-related mechanical strains) are varying linearly in the thickness direction. This provides the motivation for decomposing the nonlinear thermal eigenstrains into a linear and a nonlinear part. The linear part results in an eigenstretch and an eigencurvature of the midplane of the plate, see Fig. 4. The eigenstretch is free to develop, because of the joints between neighboring pavement plates. The eigencurvature, in turn, is constrained by the Winkler foundation on which the plate is resting. Thus, the eigencurvature results in thermal stresses of the plate. These stresses, however, turn out to be negligible relative to the thermal stresses resulting from the nonlinear part of the eigenstrains [4]. This nonlinear part of the eigenstrains can be interpreted as an eigendistortion of the generators of the plates. The latter are prevented at the scale of the plate generators, because they must remain straight according to Kirchhoff’s normal hypothesis. Thus, stress-related mechanical strains are activated, which have the same size and distribution, but the opposite sign, as the nonlinear eigenstrains. The mechanical strains result in self-equilibrated thermal stresses, see Fig. 5, and note the similarity with the distribution of the eigendistortion illustrated in Fig. 4.

Multiscale structural analysis continues with quantifying the average microscopic strains of the cement paste and of the aggregates, based on their thermal eigenstrains and the macroscopic strain of concrete $\mathbf {E}_\mathrm{con}$, using the following concentration–influence relation [23]:

12 $$\begin{aligned} \varvec{\varepsilon }_{k} = \mathbb {A}_k : \mathbf {E}_{\hom } + \sum _{j = {\hbox {cp}}, {\hbox {agg}}} \mathbb {D}_{kj} : \varvec{\varepsilon }_j^e, \quad k \in [{\hbox {cp}}, {\hbox {agg}}] \end{aligned}$$

where $\mathbb {D}_{kj}$ denotes the eigenstrain influence tensor describing the influence of the eigenstrain of material phase *j* on the total strain of material phase *k*. The average microscopic stresses of the cement paste and of the aggregates follow from the phase-specific versions of the generalized Hooke’s law: $\varvec{\sigma }_{k} = \mathbb {C}_{k}:(\varvec{\varepsilon }_{k} - \varvec{\varepsilon }_{k}^e)$. The microstresses of the cement paste differ from those of the aggregates, because the two material phases have different elastic stiffnesses and different coefficients of thermal expansion [22]. This raises the interest in quantifying the stresses transmitted across the interfacial transition zones (ITZs) separating the cement paste matrix from the aggregates. The microstress states of the ITZs can be quantified based on the known stress and strain states of the aggregates, continuity conditions regarding traction vectors and displacements across the interface between the aggregates and the surrounding ITZ, as well as the elasticity law of the interfaces, see [4, 24] for details. These developments establish the basis required for future sensitivity analysis regarding the influence of the thermal expansion coefficient of cement paste on the thermal stresses of pavement plates subjected to transient heat conduction.

### Implications for civil engineering design

Results from fundamental research provide an interesting view on a durability issue. The described multiscale poromechanics model allows for identification of the water uptake or release process of hydration products when cooled down or heated up. This nanoscopic material phenomenon governs the strong dependence of the macroscopic thermal expansion coefficient of the cement paste on its internal relative humidity. Within the regime of intermediate relative humidity, the thermal expansion coefficient of the cement paste is much larger than that of the aggregates, which gives rise to self-equilibrated internal stresses, as the temperature is changing, see [22] for details. In view of daily cycles of temperature and relative humidity, these self-equilibrated thermal stresses represent a fatigue problem. It can be a serious threat to the long-term integrity and durability of concrete. This provides high motivation for future research on this important topic.

The temperature inside pavement plates subjected to transient heat conduction is distributed nonlinearly in the thickness direction. State-of-the-art guidelines for quantification of thermal stresses of pavement plates recommend the assumption of an “equivalent” linear distribution of the temperature along the thickness of the plate, see, e.g., [5]. The presented example underscores that this assumption is unjustified, because the thermal stresses are governed by the nonlinear part of the temperature distribution [4]. The assumption of an “equivalent” linear temperature distribution was also shown to be unnecessary, because the analysis of a nonlinear temperature distribution is straightforward. At first, the spatially nonlinear temperature changes are multiplied with the thermal expansion coefficient of concrete, in order to compute the corresponding eigenstrains. The latter are subdivided into two parts: (i) the linear part, associated with an eigenstretch and an eigencurvature of the midplane of the plate, and (ii) the nonlinear part, referring to an eigendistortion of the generators of the plate. According to Kirchhoff’s normal hypothesis, these eigendistortions are prevented at the scale of the plate generators. This activates self-equilibrated thermal stresses which can be quantified in a straightforward fashion.

## High-dynamic strength of specimens made of cementitious materials

Important infrastructure must not only withstand regular service loads but also exceptional loads. Such infrastructure includes, but is not restricted to, schools, hospitals, power plants, tunnels, and bridges subjected to impact and blast loads. These dynamic loads may result, e.g., from cars accidentally crashing into engineering structures, or from detonation of so-called Improvised Explosive Devices. This provided the motivation for fundamental scientific research regarding the high-dynamic strength of specimens made of cementitious materials.

**Fig. 6:**
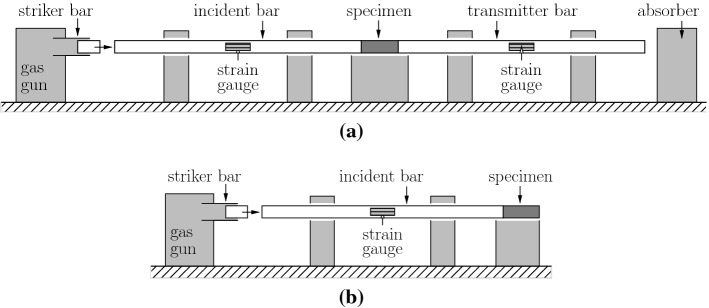
Setup of split Hopkinson pressure bars for testing of high-dynamic strength: **a** compression, and **b** tension

**Fig. 7:**
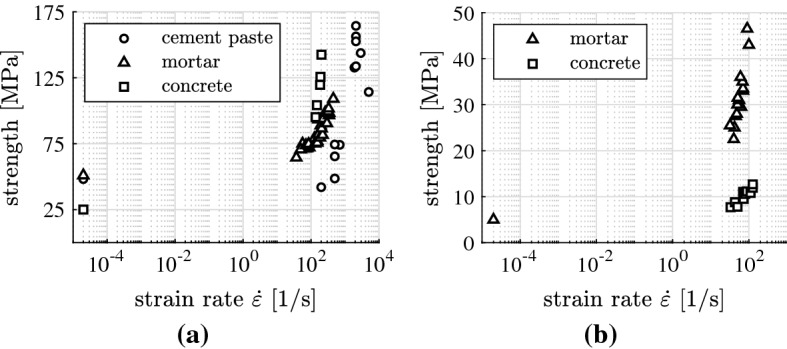
Results from quasi-static and high-dynamic testing of specimens made from cementitious materials: **a** compressive strength values [6, 26, 27], and **b** tensile strength values [28, 29]

Split Hopkinson Pressure Bars represent a popular test equipment for the experimental determination of high-dynamic strength values. As for high-dynamic compression tests, the setup consists of the serial arrangement of a striker bar, an incident bar, a cylindrical specimen, a transmitter bar, and an adsorber, see Fig. 6a. The striker bar is shot with a gas gun against the incident bar. The impact results in a compression pulse propagating along the incident bar. When the pulse strikes the interface with the specimen, it is partly transmitted and partly reflected. The transmitted compression pulse propagates along the specimen. When it strikes the interface with the transmitter bar, it is again partly transmitted and partly reflected. The stress and strain histories experienced by the specimen can be computed by means of readings of strain gauges mounted to the incident and transmitter bars [25]. This allows for quantitative determination of the high-dynamic compressive strength, sustained by the specimen. The focus of this work is on tests of cement paste cylinders by Fischer et al. [6], mortar cylinders by Zhang et al. [26], and concrete cylinders by Kühn et al. [27], see Fig. 7a.

As for high-dynamic tension tests, the setup of a Split Hopkinson Pressure Bar is limited to a striker bar, an incident bar, and a specimen, see Fig. 6b. Testing starts, analogous to high-dynamic compression tests, with shooting the striker bar against the incident bar. The compression pulse transmitted to the specimen is entirely reflected at the free end of the specimen. This creates a tension pulse propagating in the opposite direction. It interferes with the rest of the incoming compression pulse. The effective stress $\sigma _{\mathrm{eff}}(x,t)$ experienced by the specimen is the sum of the incoming compression pulse $\sigma _{\mathrm{comp}}(x,t)$ and the reflected tension pulse $\sigma _{\mathrm{tens}}(x,t)$,

13 $$\begin{aligned} {\sigma _{\mathrm{eff}}(x,t)=\sigma _{\mathrm{comp}}(x,t)+\sigma _{\mathrm{tens}}(x,t)\,,} \end{aligned}$$

where *x* denotes the position and *t* the time variable. Regarding quantification of the maximum tensile stress experienced by the specimen, different evaluation procedures are used, see, e.g., [28, 29]. They are based on formulae from the theory of wave propagation through isotropic elastic media. The focus of the present paper lies on tests of mortar cylinders by Brara and Klepaczko [28] and concrete cylinders by Erzar and Forquin [29], see Fig. 7a.

### Results from recent fundamental research

The high-dynamic strength $f_{\mathrm{dyn}}$ is traditionally quantified by multiplying the quasi-static strength $f_{\mathrm{sta}}$ with the Dynamic Strength Increase Factor (DIF)

14 $$\begin{aligned} {f_{\mathrm{dyn}} = f_{\mathrm{sta}}\cdot \text {DIF}}\, . \end{aligned}$$

**Fig. 8:**
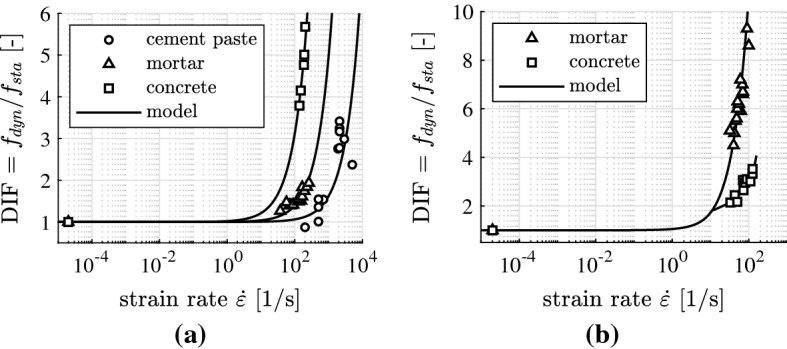
DIF values as a function of the strain rate; points refer to experimental data taken from Fig. 7, solid lines to the developed engineering-mechanics model: **a** compression tests analyzed in [3, 6, 7], and **b** tension tests analyzed in [8]

This dimensionless quantity is equal to the strength value obtained for a specific speed of loading, divided by the quasi-static strength of the tested material, see Fig. 8 for DIF values obtained from the strength values in Fig. 7.

In order to explain the high-dynamic strength gain, many models accounting for rate-dependent material properties and/or inertial confinement were proposed. Rate-dependent models are based on the assumption that the mechanical properties of cementitious materials depend on the loading rate, see, e.g., [30, 31] for the high-dynamic strength gain in compression and in tension, respectively. Models accounting for the inertial confinement consider that triaxial stress states develop even under high-dynamic uniaxial loading. Under high-dynamic uniaxial compression, triaxial compressive stress states are envisaged. Triaxial compression increases the strength of cementitious materials relative to their uniaxial compressive strength. This is sufficient for describing the high-dynamic strength gain, see, e.g., [32]. Under high-dynamic uniaxial tension, in turn, triaxial tensile stress states are envisaged. Triaxial tension decreases the strength of cementitious materials relative to their uniaxial tensile strength. This leads to a high-dynamic strength *loss*, see, e.g., [33]. In order to overcompensate this effect, such that the experimentally observed strength *gain* can be modeled, rate-dependent material properties are introduced, see, e.g., [34]. The discussed models have in common that some of the input parameters cannot be predicted. Thus, they must be identified such that test data are reproduced in the best-possible fashion. This provided the motivation for fundamental research on the present topic. It is aimed at developing better formulae, characterized by reducing the number of fitted parameters to a minimum.

An engineering-mechanics model for the high-dynamic strength was developed by Fischer et al. [6] for compression tests and extended by Binder et al. [8] for tension tests. The elasto-brittle model is based on the following hypotheses:

- Also for high-dynamic loading, cracking will start when the quasi-static strength of the material is reached.
- Cracks are assumed to propagate at a speed that is virtually equal to the velocity of shear waves.
- High-dynamic strengthening occurs during the failure process, lasting from the start of crack propagation to disintegration of the specimen.

**Fig. 9:**
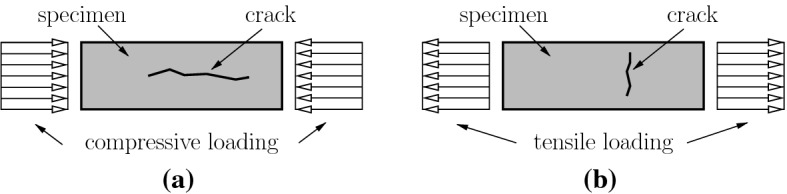
Predominant direction of the crack propagation in **a** a high-dynamic compression test, and **b** a high-dynamic tension test

The model of Fischer et al. [6] for high-dynamic compressive strength is based on the following processes. The first crack nucleates at the point where the minimum of the strength prevails. The crack starts to propagate in the loading direction, see Fig. 9a. Because the material on both sides of the crack remains intact, the axial normal stresses inside the specimen can further increase. This results in additional cracks, nucleating at other points inside the specimen. They also propagate predominantly in the direction of loading. The specimen disintegrates when the first crack has propagated through the entire specimen.

The conceptual simplicity of the described model allowed for deriving the following analytical expression for the DIF [6]:

15 $$\begin{aligned} \mathrm {DIF}^{{\text {mod}}} = 1 + \frac{E\,\dot{\varepsilon }}{f_{c}}\,\frac{\ell }{\sqrt{\mu /\varrho }} \end{aligned}$$

where *E* denotes Young’s modulus, $\dot{\varepsilon }$ the strain rate, $f_{c}$ the quasi-static uniaxial compressive strength, $\ell$ the length of crack propagation, $\mu$ the shear modulus, and $\varrho$ the mass density. Thus, $E\,\dot{\varepsilon }$ represents the stress rate, $\sqrt{\mu /\varrho }$ denotes the speed of shear waves, which is virtually equal to the speed of crack propagation [6], and $\ell /\sqrt{\mu /\varrho }$ stands for the duration of the failure process, lasting from the start of crack propagation to disintegration of the specimen.

**Fig. 10:**
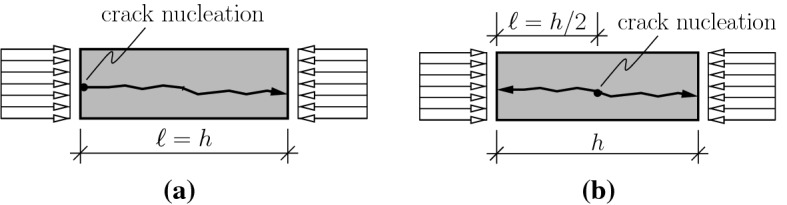
Bounds of the length of crack propagation, $\ell$, in a compression test: **a** upper bound, and **b** lower bound

The input quantities *E*, $f_{c}$, $\mu$, and $\varrho$ can be quantified by means of standard testing methods. The quantity $\ell$, however, is associated with a degree of uncertainty, because the position at which the first crack nucleates cannot be predicted. Still, bounds for $\ell$ can be derived as follows [6]:

1. If the first crack nucleates at one of the interfaces between the specimen and the load plates, the propagating crack edge has to travel along the total axial length *h* of the specimen. Thus, the upper bound for $\ell$ is equal to *h*, see Fig.  10a.
2. If the crack nucleates at the center of the specimen, two crack edges are propagating simultaneously. Each of them has to cover a distance equal to *h*/2. Thus, the lower bound for $\ell$ is equal to *h*/2, see Fig.  10b.

Consequently, realistic values of $\ell$ must be located in the following interval:

16 $$\begin{aligned} 0.50\,h \le \ell \le 1.00\,h\,. \end{aligned}$$

**Fig. 11:**
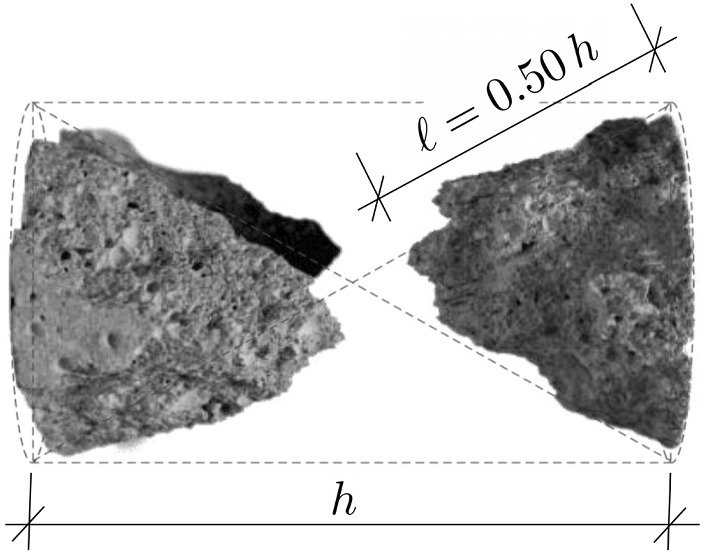
Photo showing fragments of specimens after high-dynamic testing by Kühn et al. [27]

A check was made whether or not high-dynamic strength values from complete testing campaigns can be reproduced, based on Eq. (15) and on one specific value of $\ell$ that satisfies (16). In more detail, $\ell$ was identified for each test series by minimizing the difference $\mathcal {E}$ between the modeled DIF values according to Eq. (15) and the experimentally determined $\text {DIF}^{\mathrm{exp}}$ values,

17 $$\begin{aligned} \mathcal {E}(\ell ) = \sqrt{ \frac{1}{n}\, \sum _{i=1}^n \Big [ \text {DIF}^{\mathrm{mod}}_i(\ell ) - \text {DIF}^{\mathrm{exp}}_i \Big ]^2 } \quad \rightarrow \quad \text {min}\,, \end{aligned}$$

where *n* denotes the number of high-dynamic tests included in the analyzed test series. The corresponding analyses focused on results from three testing campaigns of cylinders, made of cement paste, mortar, and concrete, respectively, illustrated as points in Fig. 8a. These three investigations have in common that the input quantities *E*, $f_{c}$, $\mu$, and $\varrho$ were provided by the experimenters. They enter Eq. (15) as known input. The crack propagation length $\ell$ was optimized according to Eq. (17). Based on $\ell = 0.59\,h$, the high-dynamic strength values of the specimens made of cement paste could be reproduced, see [6] and Fig. 8a. The high-dynamic strength values of the specimens made of mortar could be reproduced for $\ell = 0.74\,h$, see [3] and Fig. 8a. Photographs showing the destroyed concrete cylinders after high-dynamic testing, see Fig. 11, taken from [27], suggest that $\ell = 0.50\,h$. Based on this value, the corresponding high-dynamic strength tests could be reproduced, see [7] and Fig. 8a.

An engineering-mechanics model for the high-dynamic tensile strength was developed by Binder et al. [8]. It represents a continuation of the previously described line of research. The elasto-brittle model is based on the theory of wave propagation through isotropic, elastic media. The following processes were envisaged to occur during a high-dynamic tension test. The first crack nucleates inside the specific cross-section of the specimen, where the effective stress $\sigma _\text {eff}$ according to Eq. (13) reaches the quasi-static uniaxial tensile strength first. The crack starts propagating inside this cross-section, orthogonal to the loading direction, see Fig. 9b. The speed of crack propagation is again virtually equal to the speed of shear waves. Stress pulses, striking the propagating crack, are reflected. Stress pulses that do not strike the crack pass through the intact part of the cross-section containing the crack. The high-dynamic tensile strength of the tested specimen is equal to the tensile stress, transmitted across the last bridge of material, right before the specimen disintegrates into two pieces. In order to assess the model, Binder et al. [8] have used it for the analysis of the high-dynamic tensile strength values of mortar and concrete cylinders illustrated as points in Fig 8b. The test results could be reproduced, see the solid lines in Fig. 8b.

The described elasto-brittle modeling approach allowed for explaining high-dynamic compression and tension tests. It was corroborated in the framework of five different testing campaigns, carried out in five different laboratories by five different teams of experimenters, using cylindrical specimens made of cement paste, mortar, and concrete.

### Implications for civil engineering design

The design of dynamically loaded structures is a challenging task. Main difficulties are the specification of the design loads and the investigation of the load-carrying behavior. Concerning the latter, practitioners prefer static to dynamic simulations. In order to account for the dynamic nature of the underlying problem, static analyses are based on increased loads and increased values of material strength. The latter are obtained by multiplying the quasi-static strength with DIF values depending on the loading rate. DIF formulae, published in the Model Code 2010 [35], enjoy great popularity, because this Model Code is the official pre-standard of the International Federation for Structural Concrete (fib).

Recent fundamental research has led to a significantly better insight into high-dynamic strength tests of specimens of cementitious materials. Also under high-dynamic loading, cracking will start when the quasi-static strength is reached. This implies that a structure will be damaged if the dynamic stress exceeds the quasi-static strength, no matter how fast the stress is increased, and no matter how short the stress pulse lasts. In addition, the five examples illustrated in Fig. 8 underline that DIF values obtained from testing with Split Hopkinson Pressure Bars depend on the size of the tested specimens. Thus, DIF values are structural properties of the tested specimens rather than material properties.

Based on the improved understanding of high-dynamic strength tests, quasi-static design approaches for dynamic problems can be scrutinized as follows. Quasi-static design calculations are to be based on the quasi-static strength of cementitious materials. The calculations are realistic provided that the stresses remain smaller than the strength. If the calculated stresses reach the quasi-static strength, the analyzed structure will be damaged at least locally. In order to gain realistic insight into the structural damage, dynamic analysis appears to be indispensable.^1^ Thereby, it is important to explicitly account for a realistic speed of crack propagation. In addition, reliable simulations require realistic crack patterns. This raises the need for advanced models, capable of predicting realistic directions of crack propagation. As regards the material stiffness and strength, in turn, it appears to be realistic to use rate-independent properties. The latter are accessible by means of standard quasi-static material testing.

## Hybrid and classical analysis of segmental tunnel rings

The linings of tunnels, excavated by boring machines, consist of segmental rings. The individual rings are assembled by precast segments. Hence, these rings contain segment-to-segment interfacial joints. The mechanical behavior of the joints is nontrivial, because of (i) nonlinearities resulting from bending-induced partial segment-from-segment separation, (ii) nonlinearities in consequence of the load-dependent material behavior of concrete and of the existence of steel bolts connecting neighboring segments, and (iii) the time-dependent viscoelastic material behavior of concrete. This renders structural analysis of segmental tunnel rings challenging.

Early analytical models for structural analysis of segmental tunnel rings were restricted to closed rings, without explicit consideration of the joints, see, e.g., [36, 37]. In order to improve these simplistic models, “correction factors” of their stiffness were provided. This enabled analytical structural investigations with “equivalent” continuous tunnel rings, see [38]. Because of its simplicity, this has become the prevailing model for design-oriented structural analysis of segmental tunnel rings.

Lee et al. [39] are pioneers who developed analytical models for structural analysis of segmental tunnel rings, with explicit consideration of the joints. They assumed that the relative rotation angles at the joints were linear functions of the internal forces transmitted across the joints. These functions were established by means of the unit force method for determination of the internal forces and the displacements resulting from both the external loading and the relative rotation angles. Blom [40] as well as El Naggar and Hinchberger [41] simplified this mode of analysis. They argued that the relative rotation angles at the joints result in rigid-body displacements of the segments. This approach allows for a reliable determination of Serviceability Limit States of segmental tunnel rings subjected to imperceptibly anisotropic ground pressure, associated with coefficients of lateral ground pressure around 0.9, see, e.g., Blom [40].

**Fig. 12:**
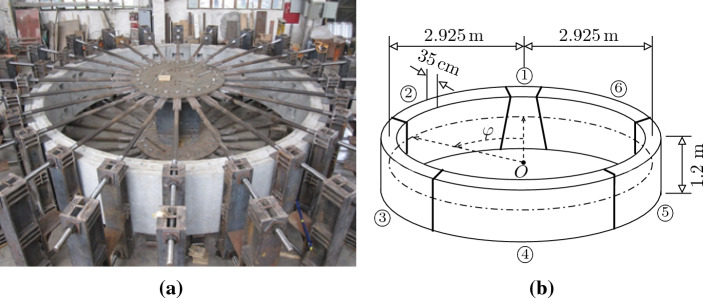
**a** Photograph and **b** geometric dimensions of a segmental tunnel ring, tested at Tongji University, according to [43] and [9], respectively

A series of real-scale bearing capacity tests of segmental tunnel rings, see, e.g., [42, 43] and Fig. 12, have provided experimental evidence that Ultimate Limit States of such tunnel rings are associated with the bearing capacity of the joints. State-of-the-art models of the joints, see, e.g., [44], suggest that the relative rotation angles increase nonlinearly with increasing loading. This provided the motivation for fundamental research, focusing on the development of methods for structural analysis of segmental tunnel rings up to their bearing capacity. These methods account for the nonlinearities resulting from (i) bending-induced cracking of the segments, (ii) segment-from-segment separation, and (iii) the nonlinear behavior of the materials of the joints.

### Results from recent fundamental research

Two types of structural analysis of segmental tunnel rings are discussed in the following. The first type refers to the hybrid analysis of a real-scale test in which a segmental tunnel ring was subjected to 24 point loads. The analysis is hybrid because the external loading *and* the measured relative rotations at the segment-to-segment interfaces are used as input. The second type of structural analysis refers to a classical analysis of a segmental tunnel ring subjected to ground pressure. The analysis is classical because the external loading is used as input, whereas the relative rotations at the segment-to-segment interfaces are computed as functions of the bending moments and normal forces transmitted across these interfaces. Both types of analysis are based on transfer relations, discussed next.

Analytical methods for structural analysis of segmental tunnels, based on transfer relations, were developed by Zhang et al. [9]. These relations represent analytical solutions of the linear theory of circular arches. The vector of the state variables at an arbitrary cross-section, defined by the angular coordinate $\varphi$, is obtained by multiplying the so-called transfer matrix by the vector of the state variables at the initial cross-section (index “*i*”), i.e., at $\varphi _i = 0$. The transfer relations read as [9]18where *u* and *v* stand for the radial and tangential component, respectively, of the displacement of the axis of the ring; $\theta$ represents the cross-sectional rotation; *M*, *N*, and *V* denote the bending moment, the axial force, and the shear force, respectively; The mathematical expressions for the nonzero elements of the transfer matrix read as [9]

19 $$\begin{aligned} T_{13}(\varphi )= & {} R~\sin \varphi \,, \displaystyle T_{14}(\varphi )=\frac{R^2}{EI}(\cos \varphi -1)\,, \nonumber \\ \displaystyle T_{15}(\varphi )= & {} \frac{R}{EA}\frac{1}{2}\varphi \sin \varphi + \frac{R^3}{EI} \left( \frac{1}{2}\varphi \sin \varphi +\cos \varphi -1 \right) , \nonumber \\ \displaystyle T_{16}(\varphi )= & {} \frac{R}{EA} \left( \frac{1}{2}\varphi \cos \varphi - \frac{1}{2}\sin \varphi \right) + \frac{R^3}{EI} \left( \frac{1}{2}\varphi \cos \varphi - \frac{1}{2}\sin \varphi \right) , \nonumber \\ T_{23}(\varphi )= & {} R~(\cos \varphi -1)\,, \displaystyle T_{24}(\varphi )=\frac{R^2}{EI}(\varphi -\sin \varphi )\,, \nonumber \\ \displaystyle T_{25}(\varphi )= & {} \frac{R}{EA} \left( \frac{1}{2}\varphi \cos \varphi + \frac{1}{2}\sin \varphi \right) +\frac{R^3}{EI} \left( \varphi - \frac{3}{2}\sin \varphi + \frac{1}{2}\varphi \cos \varphi \right) , \nonumber \\ \displaystyle T_{26}(\varphi )= & {} \frac{R}{EA} \left( - \frac{1}{2}\varphi \sin \varphi \right) +\frac{R^3}{EI} \left( 1 - \cos \varphi - \frac{1}{2}\varphi \sin \varphi \right) , \nonumber \\ \displaystyle T_{34}(\varphi )= & {} -\frac{R}{EI}\varphi \,, \displaystyle T_{35}(\varphi )=\frac{R^2}{EI}(\sin \varphi -\varphi ) \,, \displaystyle T_{36}(\varphi )=\frac{R^2}{EI}(\cos \varphi -1) \,, \nonumber \\ T_{45}(\varphi )= & {} R~(1-\cos \varphi )\,, T_{46}(\varphi )=R~\sin \varphi \,. \end{aligned}$$

The top six elements in the last column of the transfer matrix contain so-called load integrals. They represent analytical solutions for dead load [9], ground pressure [45], uniform temperature changes [46], point loads [9], and discontinuities of the kinematic variables at the joints [9]. These relations serve as the vehicle for structural analysis of segmental tunnel rings.

A specific mode of such analysis is a hybrid mode. In the given context, it refers to re-analysis of a real-scale bearing capacity test, carried out at Tongji University, see Fig. 12. The ring was subjected to anisotropic loading, imposed by 24 hydraulic jacks. The vertical and horizontal convergences and the relative rotation angles at the joints were measured. Hybrid structural analysis of the tested ring is based on two types of input: prescribed point loads and measured relative rotation angles at the joints. The proposed approach for such an analysis follows the scientific work by Blom [40] and by El Naggar and Hinchberger [41]. They assumed that the relative rotation angles at the joints result in rigid-body displacements of the segments. This allows for subdividing the hybrid analysis into two load cases [10].

**Fig. 13:**
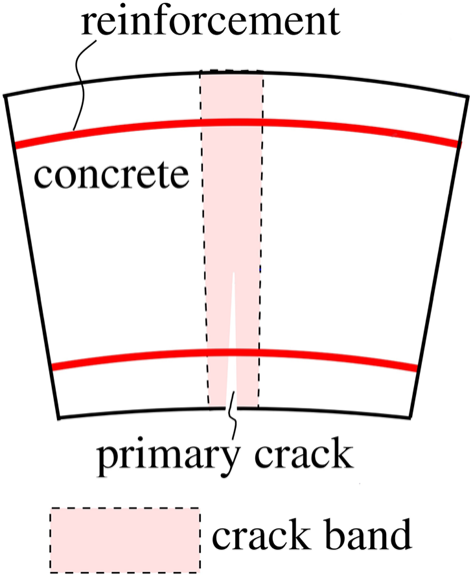
Characteristic element between two cracks for the hybrid analysis with a central crack band between undamaged zones

**Fig. 14:**
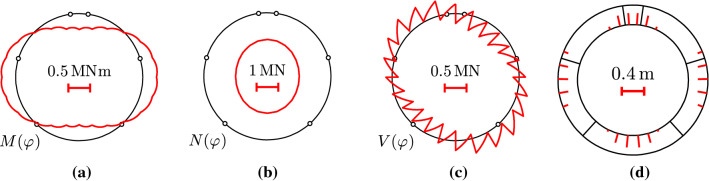
Analysis results: distributions of **a** bending moments, **b** axial forces, and **c** shear forces; **d** depths of cracks of segments, associated with the bearing capacity [10]

Load case I refers to the point loads. The relative rotation angles are set equal to zero. Thus, the corresponding structural analysis is one of a closed ring without joints, subjected to point loads. In this part of the hybrid analysis, bending-induced tensile cracking of the segments is considered. The segments are subdivided into elements, the characteristic size of which is equal to the distance of neighboring cracks. The elements contain a central crack band and two adjacent undamaged zones, see Fig. 13. The state of damage in the crack bands is determined by means of a multiscale model for tensile softening of concrete [47]. Subsequently, the effective bending and extensional stiffnesses of the damaged element are quantified, using the Voigt-Reuss-Hill estimate. The effective stiffnesses of the elements are used for simulations of the segmental tunnel ring, with element-wise constant values of effective bending and extensional stiffnesses. The results from analysis of load case I are the final results from nonlinear hybrid analysis of the segmental tunnel ring, as far as the internal forces and the state of damage of both the segments and the joints are concerned. Figure 14 shows the distributions of the internal forces and of cracks with different depths, associated with the bearing capacity.

Load case II refers to the relative rotation angles at the joints. They are estimated from monitoring data, recorded during the test. The estimation is based on the Bernoulli-Euler hypothesis. Since the validity of this hypothesis is questionable for neck-like joints, the estimated relative rotation angles are post-processed such as to refer to rigid-body displacements of the segments. This includes symmetrization and adding the smallest possible increments such that rigid-body displacements are obtained, see [10] for details. Finally, the two load cases are superimposed. This is admissible, even though the analysis of load case I is nonlinear, because superposition of load case II only adds rigid-body displacements, and the equilibrium of the structure is formulated in the undeformed configuration [10].

**Fig. 15:**
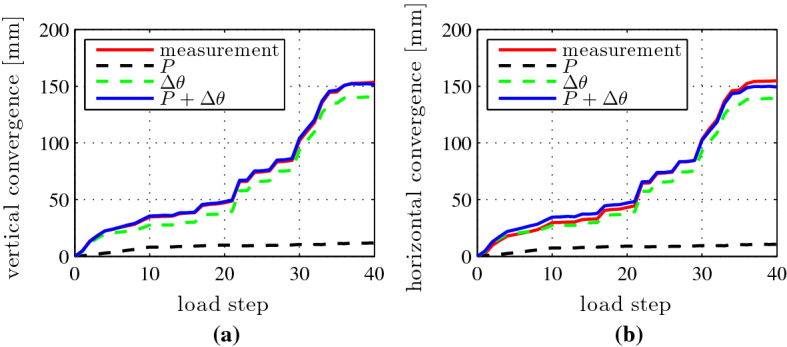
Comparison of the convergences obtained from simulation and measurements: **a** vertical convergence; **b** horizontal convergence [10]

The simulated convergences agree well with the experimental results, see Fig. 15. This underlines the usefulness of the chosen simulation strategy. Analyzing the contributions of the two load cases to the overall convergences reveals that rigid-body displacements of the segments account for approximately 95% of the convergences, whereas the deformations of the segments only account for the remaining 5%. Thus, the relative rotation angles at the joints govern the convergences of the analyzed segmental tunnel ring. Because cracking of the segments does not contribute significantly to the convergences, load case I could have been based on the assumption of linear-elastic behavior of the segments.

In general, measured relative rotation angles at the segment-to-segment interfaces are not available. Thus, the described hybrid method of analysis of segmental tunnels is not applicable. As a remedy, one needs to resort to a classical mode of analysis of such tunnels. To this end, linear transfer relations are combined with a nonlinear interface law. The latter allows for computing the relative rotation angles at segment-to-segment interfaces as a function of the bending moments and the normal forces transmitted across these interfaces. The used interface law accounts for (i) bending-induced partial segment-from-segment separation, (ii) compressive crushing of the concrete, and (iii) tensile yielding of the steel bolts connecting neighboring segments. Both the developed interface model and the corresponding method for structural analysis of segmental tunnel rings were validated by comparing computed results with measurements from independent bearing capacity tests of bolted joints and of a segmental tunnel ring, see [45].

**Fig. 16:**
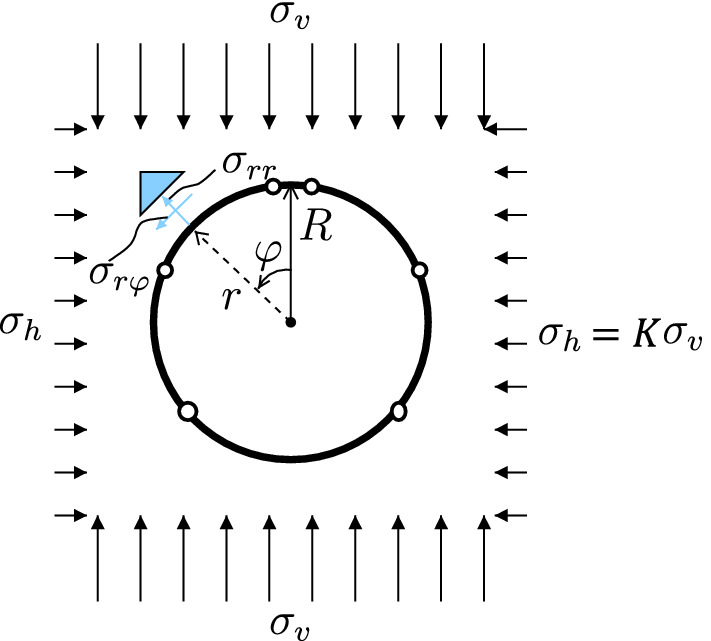
Illustration of a segmental tunnel ring subjected to ground pressure [45]

In the following, two segmental tunnel rings subjected to ground pressure (Fig. 16) are analyzed in a classical fashion, in order to compute serviceability limit states and bearing capacities. The difference between the two analyzed structures is that the first ring contains unreinforced joints, whereas the second ring contains bolts connecting neighboring segments across the joints. Herein, serviceability and ultimate limit states are defined as follows. The serviceability limit will be reached, if either the maximum stress of concrete reaches the compressive strength, or the steel of a bolt starts to yield, or the bending-induced separation of an unreinforced joint extends across more than half of the initial contact area putting the position stability of the interfaces at risk [11], or if two of these criteria are fulfilled simultaneously. The bearing capacity will be reached, if the structure develops a kinematic mechanism. As regards the investigated symmetric tunnel rings consisting of six segments, subjected to symmetric ground pressure, this mechanism is associated with the development of plastic hinges at two pairs of interfaces [45].

**Fig. 17:**
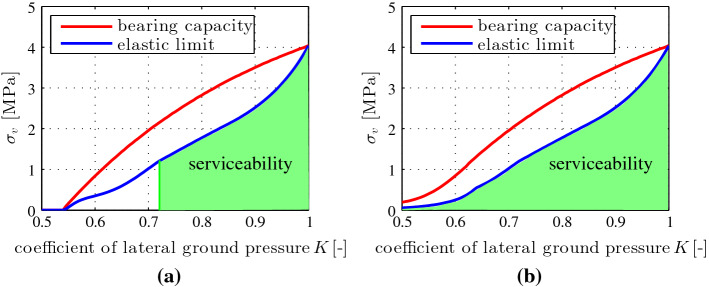
Comparison of intensities of the ground pressure related to the elastic limit and to the bearing capacity of a segmental tunnel ring with **a** unreinforced interfaces and **b** bolted interfaces [45]

A sensitivity analysis with respect to the coefficient of lateral ground pressure is performed in the interval

20 $$\begin{aligned} K \in [0.5; \,1.0], \end{aligned}$$

see Fig. 17. Results obtained for the ring with unreinforced interfaces are discussed first, see Fig. 17**a**. In this case, *K* must be larger than 0.54. Otherwise the transfer of compressive forces across the unreinforced interfaces of the segments is not sufficient to provide the normal force and the bending moment required for overall structural equilibrium, i.e., the structure is kinematically independent of the intensity of the ground pressure. In the interval $K \in [0.54; 0.72]$, bending-induced separation at some of the interfaces extends across more than half of the initial contact area, again independent of the intensity of the ground pressure. Thus, serviceability of the structure is only achieved in case of $K>0.72$, see Fig. 17a. With increasing value of *K*, both the serviceability limit and the bearing capacity increase. The discussion continues with the results obtained for the ring with bolted interfaces, see Fig. 17b. Interfacial bolts result in significant structural advantages for $K<0.72$, because the bolts ensure the position stability of the joints, compare Fig. 17a, b. As for the analyzed ring, interfacial bolts are strongly recommended for $K < 0.72$, and they are required for $K<0.54$. The latter case refers, e.g., to usual soft ground conditions.

### Implications for civil engineering design

In order to assess the safety of segmental tunnel linings in service, it is highly desirable to estimate their internal forces and the external ground pressure acting on their inaccessible outer surface. Typically, the convergences of the linings are known. They serve as input for this assessment. The reported hybrid analysis has shown that the convergences of the investigated segmental tunnel ring are governed by rigid-body displacements of the segments. Hence, there is no unique solution for the internal forces and the external ground pressure, if only the convergences are known. In this case, it is impossible to estimate the loading of segmental tunnel linings.

In tunnel engineering, serviceability limit states (SLS) and ultimate limit states (ULS) of segmental tunnels are typically related to the convergences. If an SLS is surpassed, the convergences may have a negative effect on the traffic in the tunnel, if an ULS is surpassed, they do have such a negative effect, because the clear diameter of the cross-section is reduced beyond a tolerable limit. The described fundamental research has shown that both the convergences and the bearing capacity of segmental tunnel rings are governed by the mechanical behavior of the joints. Hence, it is indispensable to account for their behavior. As regards the prediction of the convergences and the bearing capacity of segmental tunnel rings, the mechanical behavior of the reinforced concrete segments is less important, and the segments may be modeled as linear-elastic. However, consideration of cracking of the segments is of great importance when it comes to the durability assessment of segmental tunnel rings.

The reported fundamental research has clarified the role of the interfacial bolts in the structural behavior of segmental tunnel rings. Such bolts result in a significant increase in the serviceability limit of segmental tunnel rings and in the bearing capacity of such rings in case of markedly anisotropic external loading.

## Serviceability and ultimate limit states of reinforced concrete hinges

Bridges and tunnels must reach the end of their estimated service life in order to ensure the sustainability of public investments into traffic infrastructure. The long-term durability of reinforced concrete bridges is strongly affected by the performance of structural hinges. They must be durable, because their repair or replacement frequently represents a great challenge. Moreover, the associated temporary loss of serviceability may result in large costs.

Integral bridge construction is an interesting approach. The idea is to build monolithic structures, where beams, columns, and the abutments are connected by means of so-called concrete hinges. They are either unreinforced or just marginally reinforced necks in reinforced concrete bridges. Proposed by Freyssinet in the 1920s, they became quite popular in Europe in the 1960s. Pioneering design guidelines were developed by Leonhardt and Reimann in 1965, see [48]. However, concrete hinges have lost their popularity at the end of the 1960s, because their long-term behavior was unclear. Nowadays, many existing integral bridges provide evidence that concrete hinges are indeed very durable structural elements. Their regained popularity does not only call for a better scientific understanding of their structural performance, but also for modern design guidelines for verification of serviceability and ultimate limit states in the framework of the semi-probabilistic design concept.

**Fig. 18:**
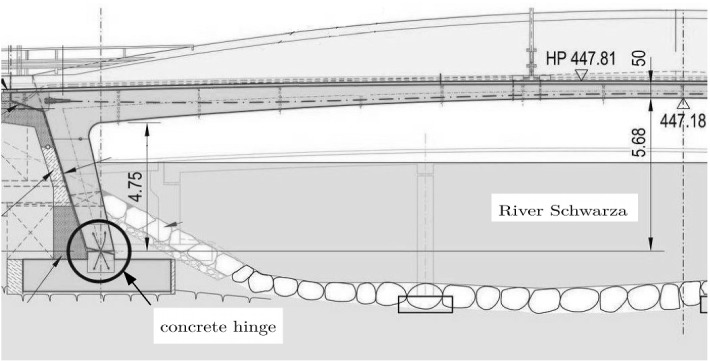
One half of the longitudinal section of a recently built integral bridge in Austria [49]

**Fig. 19:**
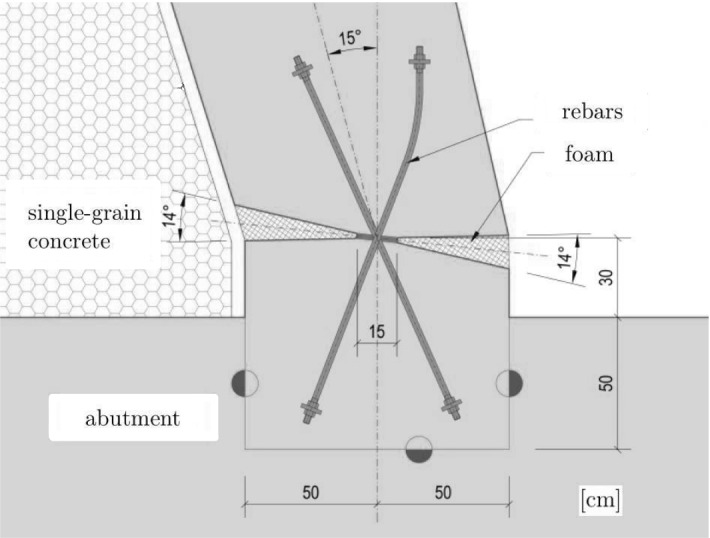
Vertical section of a concrete hinge of the integral bridge [50] shown in Fig. 18

Development of such guidelines and their application to a recently built integral bridge in Austria are the topics of this part of the present work. Figure 18 [49] shows one half of the longitudinal section of the bridge. Figure 19 illustrates a vertical section of one of the concrete hinges of the integral bridge shown in Fig. 18. It contains the reinforcement crossing the neck [50].

In the past, the structural performance of reinforced concrete hinges was, in general, investigated experimentally, using a test protocol consisting of two steps. At first, a specific compressive normal force was applied and kept constant thereafter. This was followed by imposing a relative rotation, which was also kept constant thereafter. The first type of loading resulted in creep of concrete and the second one in stress relaxation. This form of viscoelastic behavior of concrete provided the motivation to carry out eccentric compression tests and to perform Finite Element (FE) simulations with state-of-the-art software, in order to gain detailed insight into the structural behavior of reinforced concrete hinges. Because the aforementioned design guidelines of Leonhardt and Reimann do not account explicitly for reinforcement bars that cross the neck centrically, an extension of these guidelines was needed.

### Results from recent fundamental research

**Fig. 20:**
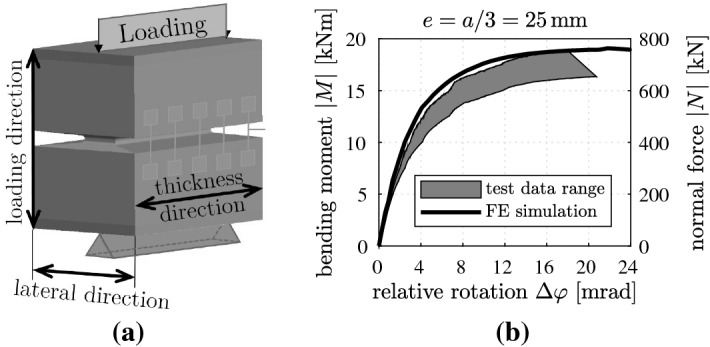
**a** Schematic illustration of the tested concrete hinges, and **b** comparison of measurements from eccentric compression tests with numerical results from three-dimensional FE simulations, see [51, 52]

Combined experimental and theoretical research was carried out in order to study the structural behavior of reinforced concrete hinges [51, 52]. The experiments included material tests of plain concrete and structural tests of three nominally identical reinforced concrete hinges. The latter were designed according to the guidelines of Leonhardt and Reimann [48]. Several types of tests were carried out. First tests were carried out using loads representative for regular service. Finally, the bearing capacity of the specimens was determined in eccentric compression tests, see [51] and Fig. 20. The measurement equipment consisted of three components. The load cell, integrated into the testing machine, recorded the normal force *N*. Multiplication by the eccentricity *e* delivered the bending moment $M = N\cdot e$. Linear Variable Displacement Transducers (LVTD) were mounted to the lateral surfaces of the specimens in order to measure the shortening, $\varDelta \ell$, and the relative rotation, $\varDelta \varphi$, of the neck, see Fig. 20. A Digital Image Correlation system was used to observe bending-induced tensile cracking along the roots of the front-side and the back-side notches.

The test results for regular service loads have shown that the magnitude of material creep of concrete is similar to the one of structural creep of reinforced concrete hinges under centric compression. Structural creep under eccentric compression, however, has turned out to be significantly larger. This is a consequence of the strong interaction between this form of creep and cracking [51]. Bending-induced macrocracks progressively open and propagate under sustained loading. Vice versa, it was concluded that stress relaxation results in progressive crack closure. This is beneficial to the long-term durability of concrete hinges [51].

As for the bearing capacity tests, the eccentricity of the normal force was set equal to one-third of the width of the neck. According to the guidelines of Leonhardt and Reimann, for this eccentricity, bending-induced tensile macrocracking will extend across one half of the initial cross-section of the neck. Along the remaining compressed ligament, the guidelines suggest a triangular stress distribution, with a vanishing normal stress at the centerline of the neck and the maximum compressive stress at its outer edge. Following this conceptual approach and setting the maximum compressive normal stress equal to the measured uniaxial compressive strength of concrete, i.e., to $f_c=46.88\,\mathrm {MPa}$, the ultimate load is expected to amount to $264\,\mathrm {kN}$. This is significantly smaller than the measured bearing capacities, amounting to $699\pm 49\,\mathrm {kN}$. Furthermore, the tested reinforced concrete hinges failed in a pronounced ductile fashion, see Fig. 20b. This was the motivation for a detailed numerical analysis of the bearing capacity tests. They were simulated using the state-of-the-art FE software "Atena science” and a nonlinear material model for concrete implemented therein [52]. Numerical simulations were carried out in order to gain quantitative insight into the stress states activated in the neck region and to find out why reinforced concrete hinges fail in such a ductile fashion.

**Fig. 21:**
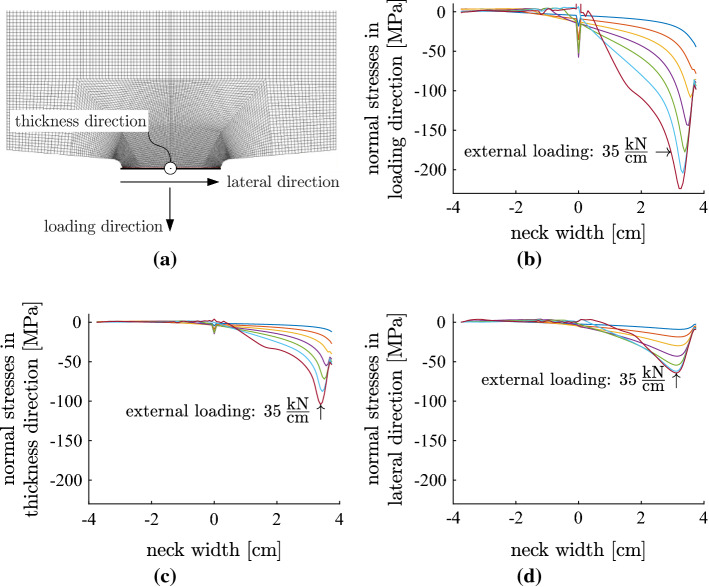
**a** Cross-section of the FE model of the concrete hinge; distribution of the normal stresses in **b** the loading direction, **c** the thickness direction, and **d** the lateral direction, for different values of external loading [52]

Results from the FE simulations have shown that triaxial compressive stress states are activated in the region of the neck [52]. Figure 21a shows a cross-section of the 3D FE discretization. Figure 21b illustrates the distribution of the normal stress in the loading direction, along the width of the neck, for different values of external loading up to 35 kN/cm. Figure 21c, d shows analogous plots of distributions of the normal stress in the thickness direction and in the lateral direction, respectively. The ratio of characteristic values of the compressive normal stresses in the three directions is 1.00 : 0.45 : 0.30, see Fig. 21 and [52]. Thus, concrete is strongly confined in the neck region. Because of the confinement pressure, both the strength and the ductility of the material are markedly larger than for uniaxial compression.

There is yet another important mechanism that enables concrete hinges to sustain large ultimate loads and to fail in a very ductile fashion. To show this, stress states at the surface of the notch, on the compressed side of the throat, are investigated. Because it is a free surface, a plane stress state prevails. A biaxial compressive stress state is the only possible stress state. The biaxial compressive strength is only slightly larger than the uniaxial compressive strength. This implies that the concrete located at the surface of the notch, on the compressed side of the throat, starts to fail rather early in a bearing capacity test of a concrete hinge. Despite this local material failure, the surface layer stays in place and, thus, does not spall. This enables the building-up of triaxial stress states inside the concrete hinges [52]. Thus, the ductile failure of concrete under biaxial compression at the surface of the neck root is the mechanism that enables concrete hinges to fail in the experimentally observed ductile fashion.

The performed numerical simulations are a good example for modern multiscale structural analysis. The first three-dimensional FE simulations were based on default input values, derived from the known Young’s modulus and the compressive strength of the concrete. The obtained numerical results overestimated both the initial stiffness and the bearing capacity of the tested concrete hinges. This confirmed one of Leonhard’s expectations that the steel rebars constrain the free autogeneous shrinkage of concrete. It promotes shrinkage-induced damage of concrete. In a classical setting, this would have called for fitting of Young’s modulus, *E*, of the tensile strength, $f_t$, and of the fracture energy, $G_f$, of concrete, such that the simulation outputs agree, in the best-possible fashion, with experimental data. Based on a multiscale model for the tensile strength and for softening of concrete [47], it was possible to reduce the number of fitted parameters from three to one. This model provides quantitative relationships between one damage variable, on the one hand, and *E*, $f_t$, and $G_f$, on the other hand. Pre-existing damage was identified in the context of correlated structural sensitivity analyses. The simulated initial stiffness agreed well with the experimental data. In order to adequately simulate the entire bearing capacity test, see Fig. 20b, the triaxial compressive strength of concrete had to be reduced from the default value proposed by the Menétrey-Willam failure criterion implemented in the state-of-the-art FE software "Atena science.” The reduced value turned out to be consistent with the regulations regarding partially loaded areas, taken from the Eurocode 2 [53]. Thus, it was concluded that the triaxial strength of concrete can be estimated reliably based on Eurocode-regulations regarding partially loaded areas [52].

Combined experimental-theoretical research has resulted in increased scientific understanding of the structural performance of reinforced concrete hinges. This provided the motivation to develop design guidelines for reinforced concrete hinges. An important aspect of these guidelines is the specification of the limit of tolerable relative rotations as a function of the compressive normal force transmitted across the neck. This is required for both serviceability and ultimate limit states of reinforced concrete hinges.

An engineering-mechanics model was the basis for the development of new design guidelines for reinforced concrete hinges in integral bridge construction. The Bernoulli–Euler hypothesis was used to describe displacement and strain states in the region of the neck. It was combined with linear-elastic and ideally plastic stress–strain relationships for concrete in compression and steel in tension. The triaxial-to-uniaxial compressive strength factor, *F*, was estimated on the basis of regulations from the Eurocode 2 [53], see above. The tensile strength of concrete was set equal to zero. The steel reinforcement was disregarded, if subjected to compression.

Besides application of the described model to integral bridge construction, it was also used for the analysis of bearing capacity tests of segmental tunnel rings. Moreover, it was applied to quantification of the behavior of segment-to-segment interfaces. These interfaces are bolted concrete hinges, see Sect. 4 and [10].

**Fig. 22:**
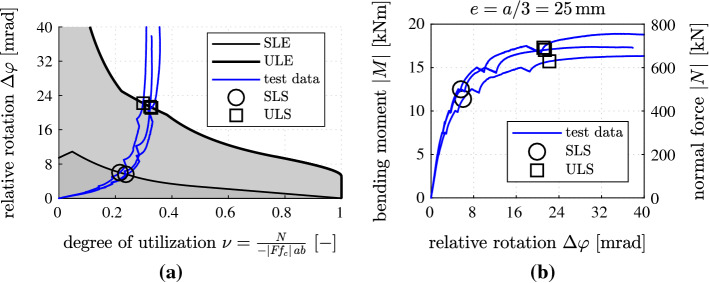
Analysis of eccentric compression tests of reinforced concrete hinges: **a** identification of serviceability and ultimate limits [11, 12]; **b** experimental data

As for integral bridge construction, analytical formulae for verification of serviceability and ultimate limits of reinforced concrete hinges were derived [11, 12]. The serviceability limit states indicate that the maximum compressive normal stress of concrete has reached the triaxial compressive strength and/or that the steel rebars have started to yield. A detailed analysis has led to consideration of four different operating conditions of reinforced concrete hinges. The obtained analytical formulae provide serviceability limits of tolerable relative rotations, $\varDelta \varphi _\ell$, as a function of the degree of utilization regarding the normal force transmitted across the hinge,

21 $$\begin{aligned} \nu = \frac{N}{-|Ff_{c}|\,ab}\,, \end{aligned}$$

where $Ff_{c}$ denotes the triaxial compressive strength of concrete and *ab* represents the cross-sectional area of the neck, see also Fig. 21a and the serviceability limit envelope (SLE) in Fig. 22a. Denoting Young’s modulus of concrete as $E_{c}$, Young’s modulus of steel as $E_s$, the reinforcement ratio as $\rho$, and the yield stress of steel as $f_y$, the SLE is based on the following analytical formulae. In “compression-dominated operation,” the entire cross-section of the neck is subjected to compressive stresses. The mathematical formulation of the SLE reads as [11]

22 $$\begin{aligned} \varDelta \varphi _{\ell }= 2\,(1-\nu )\,\frac{|Ff_{c}|}{E_{c}}\,, \qquad \nu \in [0.50;~1.00]. \end{aligned}$$

In the operating condition “tensile macrocracking up to one half of the width of the neck,” the reinforcement is subjected to compressive stresses. The mathematical formulation of the SLE reads [11]

23 $$\begin{aligned} \varDelta \varphi _{\ell }=\frac{1}{2\,\nu }\,\frac{|Ff_{c}|}{E_{c}}\,, \qquad \nu \in [0.25;~0.50]. \end{aligned}$$

In the operating condition “tensile macrocracking beyond one half of the width of the neck, resulting in tensile loading of the reinforcement,” the mathematical formulation of the SLE reads [11]

24 $$\begin{aligned} \varDelta \varphi _{\ell }= \frac{|Ff_{c}|}{\rho \,E_{s}}\left[ \left( \frac{\rho \,E_{s}}{E_{c}}-\nu \right) + \sqrt{ \left( \frac{\rho \,E_{s}}{E_{c}}-\nu \right) ^2 + \frac{\rho \,E_{s}}{E_{c}} } \right] , \qquad \nu \in [\nu ^*;~0.25], \end{aligned}$$

with

25 $$\begin{aligned} \nu ^* = \frac{1}{4} \left( \frac{f_{y}}{|Ff_{c}|}\,\frac{E_{c}}{E_{s}}+1 \right) ^{-1} - \frac{\rho \,f_{y}}{|Ff_{c}|}\,. \end{aligned}$$

Finally, in “tension-dominated operation,” the mathematical formulation of the SLE reads [11]

26 $$\begin{aligned} \varDelta \varphi _{\ell }= & {} \displaystyle \frac{2}{E_{c}} \bigg \{ f_{y} \left( \frac{E_{c}}{E_{s}}+2\,\rho \right) + 2\,|F f_{c}|\,\nu \nonumber \\&\displaystyle + \sqrt{ \left[ f_{y} \left( \frac{E_{c}}{E_{s}} + 2\,\rho \right) + 2\,|F f_{c}|\,\nu \right] ^2 - \left( f_{y}\,\frac{E_{c}}{E_{s}} \right) ^2 } \bigg \}\,, \qquad \nu \in \left[ \,-\frac{\rho \,f_{y}}{|Ff_{c}|}~;~\nu ^*\,\right] . \end{aligned}$$

The ultimate limit states reveal that the maximum compressive normal strain of concrete and/or the maximum tensile normal strain of the steel rebars has reached the corresponding ultimate limit strain. A detailed analysis has led to six different operating conditions of reinforced concrete hinges [12]. The obtained analytical formulae provide ultimate limits for $\varDelta \varphi _\ell$ as a function of $\nu$, see the ultimate limit envelope (ULE) in Fig. 22a.

Graphs, illustrating the test data, see Fig. 22b, were added to the diagrams in Fig. 22a, containing the SLE and the ULE. Because the experiments were eccentric compression tests, the bending moment *M* is directly proportional to the normal force *N*. Thus, the relation between the relative rotation $\varDelta \varphi$ and *M* is affine to the relation between $\varDelta \varphi$ and $\nu$, compare Fig. 22a, b. The points, at which the graphs of the experimental data intersect the ones of the serviceability and ultimate limit envelopes, represent pairs of limit state values, consisting of a specific normal force and a relative rotation, see the circles and squares in Fig. 22a. Marking these points in the graphs of the experimental data, see Fig. 22b, allows for identifying serviceability limit states (SLS) and ultimate limit states (ULS) of the tested concrete hinges. Up to the identified serviceability limits, the concrete hinges behave in a moderately nonlinear fashion. Significant nonlinearities occur beyond the serviceability limits, see Fig. 22b. Beyond the identified ultimate limits, the bending moment can no longer be increased significantly. The ultimate limits are nonetheless conservative, because the relative rotation may be increased in the experiment to significantly larger values, see Fig. 22b. As mentioned previously, the new design guidelines were successfully applied to a recently built integral bridge in Austria.

### Implications for civil engineering design

Combined experimental-theoretical research has enabled the development of new design guidelines for verification of serviceability and ultimate limit states of reinforced concrete hinges. Fundamental research has served its ultimate purpose to promote progress in civil engineering design. This was achieved in the framework of interdisciplinary research, carried out in the fields of integral bridge construction and mechanized tunneling, respectively.

The new design guidelines explicitly account for the crossing steel rebars which run across the neck. The rebars provide the required position stability in case bending-induced macrocracking extends beyond one half of the width of the neck.

There are interesting similarities and differences regarding serviceability and ultimate limit states of concrete hinges in the construction of integral bridges and of segmental tunnel rings. Serviceability limit states of concrete hinges in integral bridge construction are associated with elastic limits of concrete and/or steel. Serviceability limit states of segmental tunnel rings, in turn, are related to convergences that have grown so large that the traffic running through the tunnel is negatively affected, because the clear diameter of the cross-section is reduced beyond a tolerable limit. In such cases, it is almost certain that several interfaces of segments have already become plastic hinges [45]. From the viewpoint of integral bridge construction, the development of the first plastic hinge already indicates an ultimate limit state. This underlines that different engineering structures require different definitions of serviceability and ultimate limit states.

## Summary, discussion, and conclusions

Scientific research, both fundamental and applied, provides answers to open questions. Consequently, state-of-the-art models are to be scrutinized regularly. If possible, they must be improved. In the present paper, results from fundamental research in the field of engineering mechanics of concrete and reinforced concrete structures were presented. Implications on engineering design were discussed.

Recurrent cycles of temperature and relative humidity are important load cases for assessing the long-term durability of concrete and reinforced concrete structures. A multiscale poromechanics model allowed for top-down identification of a formerly unknown material phenomenon. Nanoscopic calcium-silicate-hydrates release water upon heating and take up water upon cooling in a quasi-instantaneous and reversible fashion. The corresponding changes of the internal relative humidity result in changes of the effective pore underpressures. This explains why the thermal expansion of the cement paste is a function of the internal relative humidity and why the thermal expansion in the range of intermediate relative humidities is virtually twice as large as in fully saturated or fully dried states. As for engineering design, the following conclusions are drawn:

- In the regime of intermediate internal relative humidities, the thermal expansion of the cement paste is virtually twice as large as the one of customary aggregates. Thus, temperature changes result in considerable self-equilibrated internal stresses.
- Concrete infrastructures exposed to the open air undergo daily cycles of internal stresses. The mentioned self-equilibrated internal stresses are the source of a fatigue problem. It puts at risk on the long-term integrity of the microstructure of concrete and, thus, the long-term durability of the material.

Exceptional loading in terms of sudden heating or cooling presents another serious load case. Sudden changes of temperature result in transient heat conduction. This is associated with time-dependent and spatially nonlinear temperature fields. Thus, also the thermal eigenstrains are distributed in a spatially nonlinear fashion. The linear part results in an eigenstrain and an eigencurvature of the midplane of plates or the axis of beams. The nonlinear part is associated with an eigendistortion of the generators of plates or the cross-sections of beams. The eigendistortion is prevented, however, because of Kirchhoff’s normal hypothesis. Thus, spatially nonlinear mechanical strains, i.e., stress-related strains, are activated. When added to the thermal eigenstrains, they yield linear total strains in the thickness direction of plates. This is compatible with Kirchhoff’s normal hypothesis. As for engineering design, the following conclusions are drawn:

- When engineering structures are subjected to transient heat conduction, thermal stresses are inevitably activated, no matter whether the structure is supported in a statically determinate or indeterminate fashion.
- The thermal eigenstresses are distributed nonlinearly along the generators of plates (or within the cross-section of beams). They neither contribute to the membrane forces (normal forces) nor to the bending moments. Thus, it is impossible to define equivalent linear temperature fields, notwithstanding that this is still a very popular approach, because it is recommended by state-of-the-art guidelines such as [5].

Exceptional loading in terms of high-dynamic compression or tension is another important problem. Results from fundamental scientific research suggest that cracking starts, also under high-dynamic loading, when the quasi-static strength of the material is reached. Cracks propagate at a speed which is virtually equal to the velocity of shear waves. High-dynamic strengthening occurs during the failure process, lasting from the initiation of crack propagation to disintegration of the specimen. The duration of the failure process depends on the size of the investigated structure. Thus, DIF values, obtained from tests carried out with a Split Hopkinson Pressure Bar, are structural properties of the tested specimens rather than material properties. As for engineering design, the following conclusions are drawn:

- Engineering structures made of concrete will be damaged provided that the dynamic stress exceeds the quasi-static strength, no matter how fast the stress is increased and how short the stress pulse lasts. Hence, conventional quasi-static analysis allows for identifying whether or not the structure will be damaged. The extent of the local damage, however, cannot be assessed.
- As for quantifying the local damage resulting from high-dynamic loading, it is indispensable to carry out dynamic simulations, to explicitly account for a realistic speed of crack propagation, and to use advanced models that are capable of predicting realistic directions of crack propagation also in case of multiaxial types of loading. This calls for a change of paradigm in civil engineering design.

Hybrid and classical simulation methods are well-suited for structural analysis of real-scale bearing capacity tests of segmental tunnel rings used in mechanized tunneling. Both types of analysis reported in this work were based on transfer relations, representing analytical solutions of the first-order theory of thin circular arches. Externally imposed loading was used as known input. As for the “hybrid” analysis, measured relative rotations at the interfaces between the segments also entered the analysis as known input. As for the “classical” analysis, however, interface laws had to be used in order to predict the relative rotations resulting from the bending moments and the normal forces transmitted across the interfaces between the segments. The simulations provided valuable insight into the structural behavior of segmental tunnel rings. As for engineering design, the following conclusions are drawn:

- Tunnel convergences are governed by rigid-body displacements of the tunnel segments. Thus, it is impossible to estimate the loading of segmental tunnel linings, based on measured convergences.
- The bearing capacity of segmental tunnel rings is associated with the development of a kinematic mechanism. It is characterized by the development of plastic hinges at four segment-to-segment interfaces. Bolted interfaces, representing reinforced concrete hinges, significantly increase the bearing capacity of segmental tunnel rings subjected to strongly anisotropic external loading.

Reinforced concrete hinges represent necks in reinforced concrete structures. Because of the throat, three-dimensional compressive stress states are activated in the region of the neck. The resulting confinement significantly increases the strength and the ductility of concrete. The triaxial compressive strength can be estimated based on regulations for partially loaded areas. New recommendations were elaborated for verification of serviceability and ultimate limit states of reinforced concrete hinges. Because the reinforcement was explicitly accounted for, the tolerable limits of the relative rotations are larger than those according to the guidelines of Leonhardt and Reimann. As for engineering design, the following conclusions are drawn.

- In order to ensure the position stability of unreinforced concrete hinges, bending-induced tensile macrocracking must be limited to one half of the width of the neck. This limitation can be discarded provided that tensile forces in centrically crossing steel rebars can stabilize the concrete hinge, even when tensile macrocracking extends beyond one half of the width of the neck.
- Elastic limits of concrete in compression and/or of steel in tension represent serviceability limit states of reinforced concrete hinges. Ultimate limit states refer to the situation that concrete reaches its ultimate limit strain in compression and/or steel attains its ultimate limit strain in tension.

There are interesting similarities as well as differences regarding serviceability and ultimate limit states of concrete hinges in integral bridge construction, on the one hand, and segmental tunnel rings in mechanized tunneling, on the other hand.

- Serviceability limit states of concrete hinges in integral bridge construction are associated with elastic limits of concrete and/or steel. Serviceability limit states of segmental tunnel rings, however, are associated with large convergences. This may have a negative effect on the traffic in the tunnel, because the clear diameter of the cross-section may be reduced beyond a tolerable limit. In such cases, it is very likely that several interfaces between the segments have already developed plastic hinges.
- From the viewpoint of integral bridge construction, already the development of the first plastic hinge signals an ultimate limit state. This underlines that different types of structures may be associated with different kinds of serviceability and ultimate limit states.

Finally, it is emphasized that the described fundamental research activities were carried out within an Austro-Chinese research project, bringing together researchers from Vienna University of Technology and from Tongji University, in Shanghai. The common language of the engineering sciences and the strong joint motivation to carry out fundamental research have not only led to valuable insight into important phenomena and processes governing the mechanical behavior of concrete and reinforced concrete structures, but also contributed to the advancement of civil engineering design.

## Footnotes

1 Realistic quasi-static calculations cannot be based on “high-dynamic strength values” obtained from multiplying the quasi-static strength with a DIF value, because the DIF value is a structural rather than a material property.
